# Analysis of the Molecular Evolution of Hepatitis B Virus Genotypes in Symptomatic Acute Infections in Argentina

**DOI:** 10.1371/journal.pone.0159509

**Published:** 2016-07-19

**Authors:** María Belén Rodrigo, Laura Noelia Mojsiejczuk, Carolina Torres, Ina Sevic, María Mora González López Ledesma, Paula Soledad Perez, María Belén Bouzas, Omar Galdame, Sebastián Marciano, Hugo Fainboim, Diego Martín Flichman, Rodolfo Héctor Campos

**Affiliations:** 1 Cátedra de Virología, Facultad de Farmacia y Bioquímica, Universidad de Buenos Aires, Argentina, Ciudad Autónoma de Buenos Aires, Argentina; 2 Unidad de Virología, Hospital de Enfermedades Infecciosas ‘‘F. Muñiz”, Ciudad Autónoma de Buenos Aires, Argentina; 3 Unidad de Hepatología, Hospital Italiano de Buenos Aires, Ciudad Autónoma de Buenos Aires, Argentina; 4 Unidad de Hepatopatías Infecciosas, Hospital de Enfermedades Infecciosas ‘‘F. Muñiz”, Ciudad Autónoma de Buenos Aires, Argentina; University of Athens, Medical School, GREECE

## Abstract

Hepatitis B virus (HBV) is a globally distributed human pathogen that leads to both self-limited and chronic infections. At least eight genotypes (A-H) with distinct geographical allocations and phylodynamic behaviors have been described. They differ substantially in many virological and probably some clinical parameters. The aim of this study was to analyze full-length HBV genome sequences from individuals with symptomatic acute HBV infections using phylogenetic and coalescent methods. The phylogenetic analysis resulted in the following subgenotype distribution: F1b (52.7%), A2 (18.2%), F4 (18.2%) and A1, B2, D3 and F2a 1.8% each. These results contrast with those previously reported from chronic infections, where subgenotypes F1b, F4, A2 and genotype D were evenly distributed. This differential distribution might be related to recent internal migrations and/or intrinsic biological features of each viral genotype that could impact on the probability of transmission. The coalescence analysis showed that after a diversification process started in the 80s, the current sequences of subgenotype F1b were grouped in at least four highly supported lineages, whereas subgenotype F4 revealed a more limited diversification pattern with most lineages without offspring in the present. In addition, the genetic characterization of the studied sequences showed that only two of them presented mutations of clinical relevance at S codifyng region and none at the polymerase catalytic domains. Finally, since the acute infections could be an expression of the genotypes currently being transmitted to new hosts, the predominance of subgenotype F1b might have epidemiological, as well as, clinical relevance due to its potential adverse disease outcome among the chronic cases.

## Introduction

The study of the origin, emergence, and spread of viral infections in human populations is one of the most active and productive areas of research in modern evolutionary biology [1]. Hence, the study of viral phylogeography and evolution is not only of historical significance, but by revealing the rules of viral evolution, it might also be possible to shed light on disease epidemiology [2].

Hepatitis B virus (HBV) has a remarkably complex evolutionary history. The intricate genetic organization based on overlapping reading frames, its DNA nature and the peculiar replication strategy are but a few factors that make the evolutionary analysis of this virus difficult [3–5]. HBV is classified into eight main genotypes (A-H) and two additional (I and J) were tentatively proposed [6,7]. In addition, the diversity of this virus is strongly geographically structured, with a variety of subgenotypes and inter-genotype recombinants exhibiting distinct geographical allocations [6,8].

The study of the genotype diversity might be helpful not only for understanding the mechanisms of disease pathogenesis, the development of biomarkers for diseases prognosis or the identification of potential therapeutic targets, but also for evaluating the evolutionary history of the virus [5].

On the clinical bases, HBV may cause self-limited and persistent infections, depending on the interplay between host immunity and viral evasion strategies [9,10]. Several studies performed on chronic HBV infections have suggested differential biological and clinical features for different genotypes and subgenotypes [9,11–15]. However, HBV virus from self-limited cases has not been extensively studied so far.

Taking into account that acute infections could be an expression of the genotypes currently being transmitted to new hosts, the aim of this work was to study the distribution and evolutionary dynamics of HBV genotypes in symptomatic acute infections in Buenos Aires city, Argentina.

## Materials and Methods

### Samples

Serum samples from 55 epidemiologically unrelated HBsAg positive subjects diagnosed with symptomatic acute HBV, admitted to Hospital Italiano (Buenos Aires, Argentina) and Hospital de Enfermedades Infecciosas “F. Muñiz” (Buenos Aires, Argentina), dating from 2000–2013, were included in this study. These samples have been reported and partially characterized previously by González López Ledesma et al. [11].

The inclusion criteria of the patients were: acute onset of hepatitis symptoms, levels of serum alanine aminotransferase (ALT) >10-fold the upper reference limit, positivity for IgM antibody to the hepatitis B core antigen (anti-HBc) and spontaneous HBsAg seroconversion within the first six months from the clinical onset of the infection. The exclusion criteria were: no consent; clinical or histological diagnosis of cirrhosis; immunosuppressed individuals (HIV positive with detectable viral loads or CD4^+^ lymphocytes lower than 200/mm^3^); oncological patients; HCV positive individuals; or insufficient follow-up for diagnosis. No fulminant cases were observed.

### HBV-DNA amplification

Briefly, DNA was extracted from 200μl of serum sample by using a High Pure Viral Nucleic Acid extraction Kit (Roche, Germany) according to manufacturer’s instructions. Six nested PCRs were performed to obtain the complete genome of the isolates (S1 Table). Samples were sequenced using an ABI3730*XL* Sequencer (Applied Biosystems, USA). Sequences were edited and aligned with BioEdit v.7.2.0 [16]. Sequences were deposited in Genbank with the accession numbers KJ843163 -KJ843218 (S2 Table).

### Phylogenetic analysis

For subgenotyping, complete genome sequences of the 55 collected isolates were complemented with 58 reference samples from different HBV genotypes (A-H) retrieved from GenBank.

Phylogenetic relationships were evaluated using the maximum likelihood (ML) methods. ML trees were obtained by using PhyML v.3.1 software [17] and the general time reversible (GTR) +I+G nucleotide substitution model, estimated using jModeltest v.2.1.4 [18] according to the Akaike Information Criterion (AIC). Robustness of the phylogenetic grouping was evaluated by bootstrapping (1000 replicates).

### Genetic characterization

In order to identify mutations with clinical or epidemiological relevance, nucleotide and deduced amino acid sequences belonging to S protein and viral polymerase of the isolates reported in this study were compared to prototype sequences for each genotype/subgenotype.

### Coalescence analysis

The complete genome sequences generated in this study were introduced in Bayesian coalescent analyses in order to study the population dynamics of different subgenotypes of the genotype F. Each subgenotype dataset was constructed and analyzed separately. They included the sequences obtained in this work (subgenotype F1b n = 32 and subgenotype F4 n = 11) and sequences from acute patients, derived from the same population and period, reported previously by Pezzano *et al*. [15] (FJ657521, FJ657523, FJ657524, FJ657522)

The temporal signal of each dataset was roughly studied through the root-to-tip method (genetic divergence to the root inferred from a ML tree against sampling time) using the Path-O-Gen v1.4 software (http://tree.bio.ed.ac.uk/software/pathogen/). Under this analysis, the correlation coefficient would indicate the amount of variation in genetic distance that is explained by sampling time and provides a measure of the clockliness of the data. This analysis showed evidence for temporal information in the datasets (subgenotype F1b: correlation coefficient = 0.47; subgenotype F4: correlation coefficient = 0.63) and low adjustment to a strict molecular clock.

The coalescent analysis was performed under the most appropriate model of base substitution for each dataset (GTR+I+G for subgenotype F1b and TIM2+G for subgenotype F4) estimated, as previously mentioned. The uncorrelated lognormal molecular clock model and the Bayesian Skyline model implemented in BEAST v.1.7.5 software package [19] were used. Analyses were run for 20 million generations (sampling every 2000 generations) and convergence was evaluated with Effective Sampling Size values higher than 200 for each parameter. Ten percent of the sampling was discarded as burn-in and acceptable mixing was visualized with the Tracer v1.5 software. Uncertainty in parameter estimates was evaluated in the 95% highest posterior density (HPD95%) interval. The posterior tree distribution was summarized with the program TreeAnnotator v.1.7.5 and the annotated maximum clade credibility tree (MCCT) was visualized with FigTree v.1.4.0 (http://tree.bio.ed.ac.uk/soft-ware/figtree/).

### Ethical considerations

This study was approved by the Ethics Committee of the Facultad de Farmacia y Bioquímica of the Universidad de Buenos Aires. Blood sample collection was conducted after written consent forms had been signed. The study was performed in accordance with provisions of the Declaration of Helsinki and Good Clinical Practice guidelines.

## Results

### Subgenotype distribution

**Fig 1:**
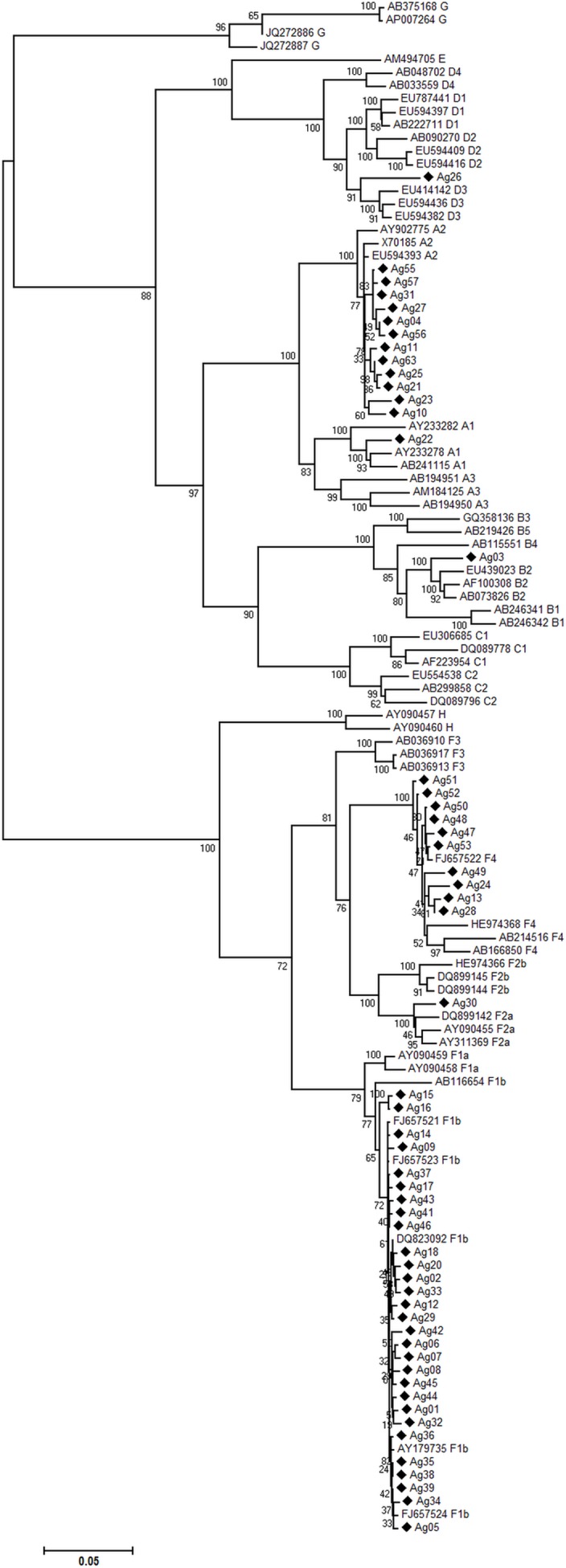
Subgenotype distribution of the acute isolates. Maximum-likelihood phylogenetic tree performed with the PhyML (v3.0) program constructed on the complete genome sequences including fifty-five sequences of symptomatic acute HBV isolates from the city of Buenos Aires (marked with ♦) and reference sequences retrieved from GenBank, indicated by their accession numbers. The numbers at each node correspond to bootstrap values obtained with 1000 replicates. The scale bar indicates the genetic distances.

In order to evaluate the subgenotype distribution, the full-length genome sequences of the 55 isolates from the acute infections were studied by phylogenetic analysis. Four out of the eight major genotypes described were present in the studied cohort. The overall subgenotype prevalence resulted as follows: F1b (52.7%), A2 (21.8%), F4 (18.2%) and A1, B2, D3 and F2a with 1.8% each one (Fig 1).

### Characterization of circulating HBsAg / Polymerase variants

The genetic characterization of the 55 sequences showed only two samples with mutations at S codifyng region (P142S, D144A in Ag10-subgenotype A2 and S143L in Ag26-subgenotype D3) in sites related to immune escape. No mutants associated with antiviral resistance were detected.

### Evolutionary analysis

**Fig 2:**
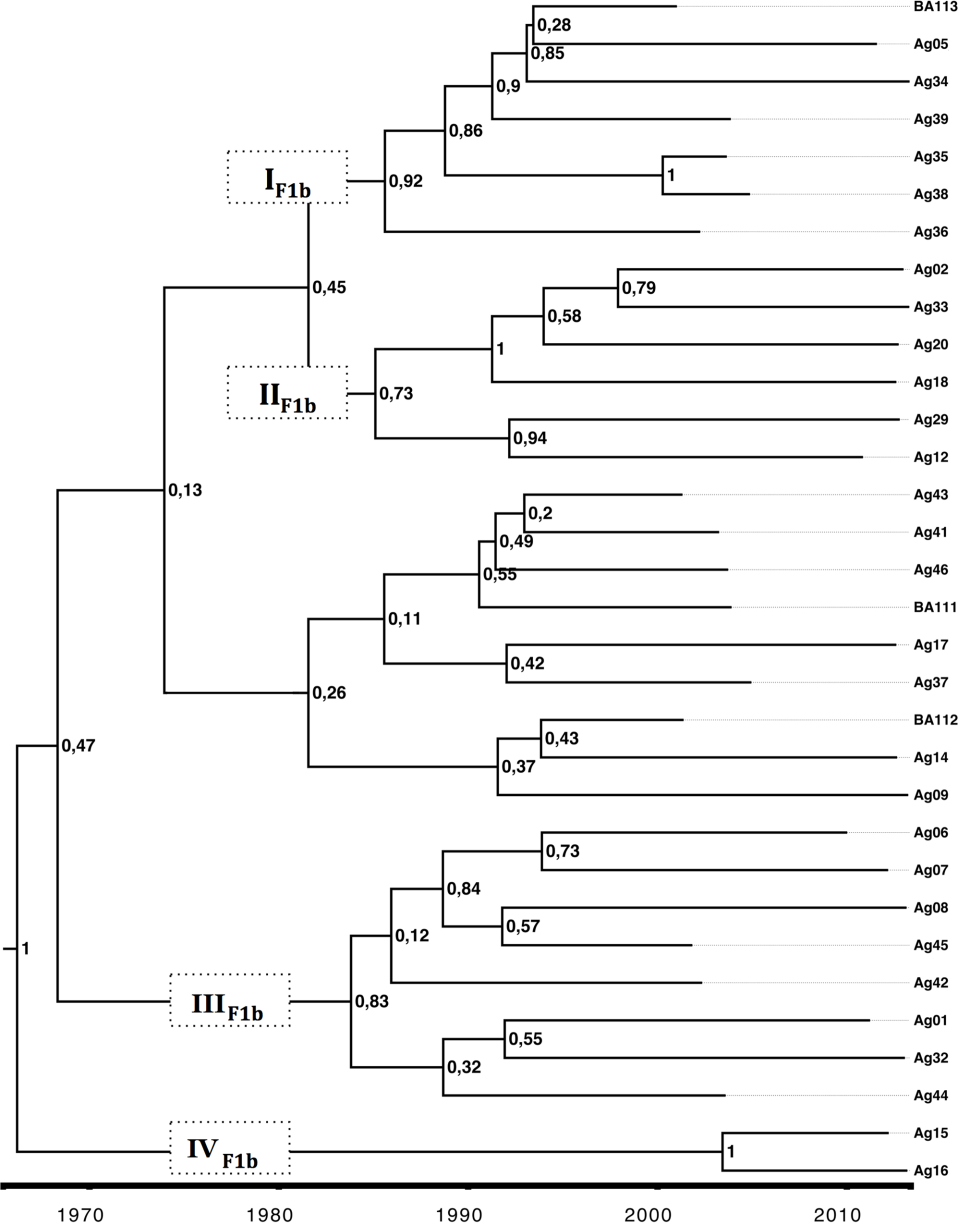
MCCT for subgenotype F1b. Maximum clade credibility tree (MCCT) performed by calibration with the terminal nodes for complete genome of acute subgenotype F1b samples. The MCCT presents for each node plots of its correspondent posterior probability. Labels I_F1b_-IV_F1b_ represent the clusters found in the circulating strains. The scale at the bottom of the tree represents time (years).

**Fig 3:**
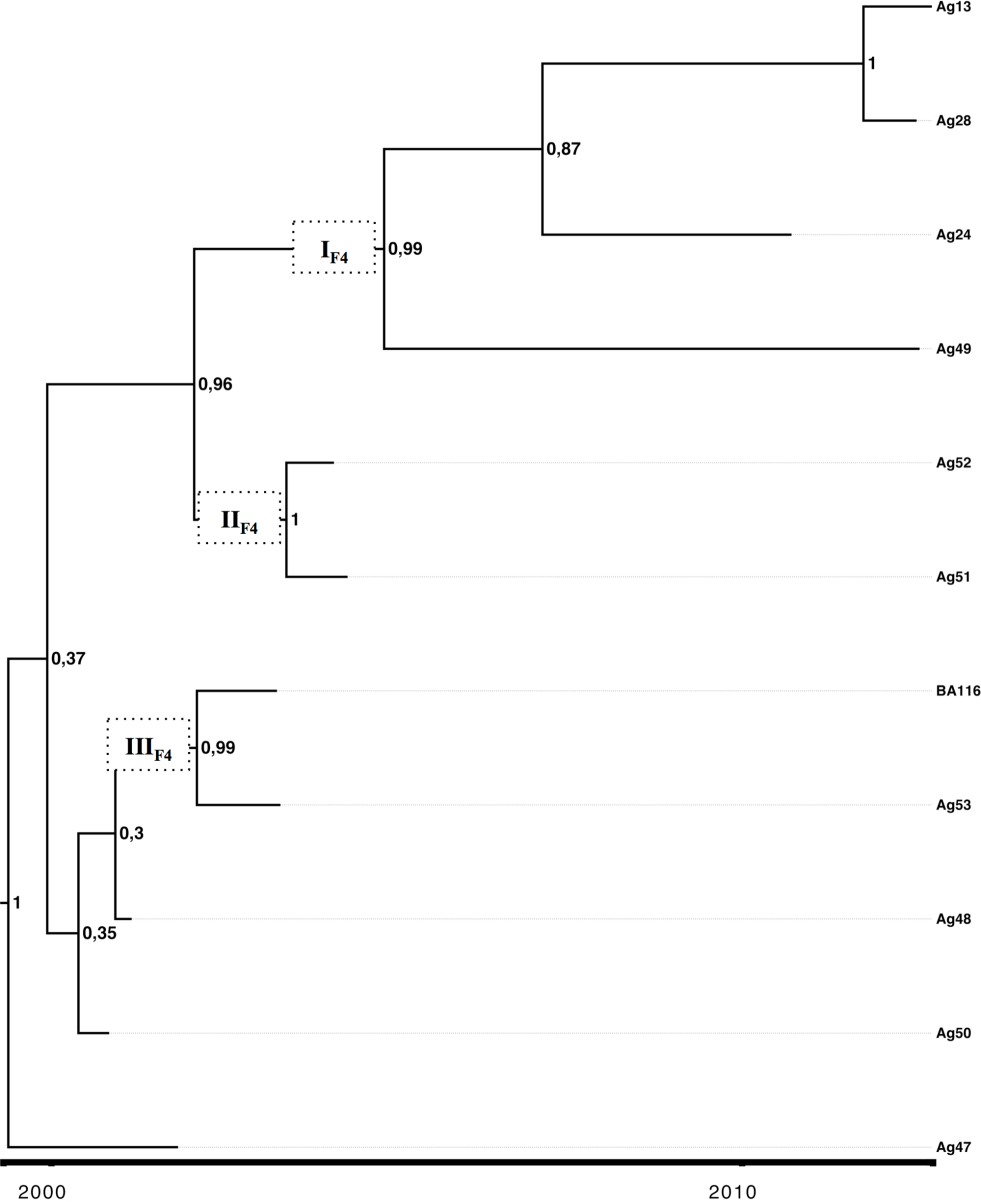
MCCT for subgenotype F4. Maximum clade credibility tree (MCCT) performed by calibration with the terminal nodes for complete genome of acute subgenotype F4 samples. The MCCT presents for each node plots of its correspondent posterior probability. Labels I_F4_-III_F4_ represent the clusters found in the circulating strains. The scale at the bottom of the tree represents time (years).

The evolutionary dynamics of isolates from acute infection by subgenotype F1b and subgenotype F4 was studied through Bayesian coalescent analyses. For subgenotype F1b and subgenotype F4, the most recent common ancestors were dated in 1966 (HPD95% = 1808–1982) and in 1990 (HPD95% = 1975–2002), respectively (Table 1). The analysis indicated that the current circulating strains from subgenotype F1b derived from at least four main lineages (groups with posterior values ≥0.70, I- IV_F1b_, in Fig 2) dated in the early 80s and that would have its diversification process during the 90s. In contrast, a different scenario was drawn for subgenotype F4, which would present a more limited diversification pattern with at least three supported lineages (I-III_F4_) but only one of them with offspring in the more recent sampling dates (I_F4_) with an ancestor dated on 2004 (Fig 3). However a cautious interpretation is needed owing to the low number of sequences available for this subgenotype.

**Table 1:** Co-estimation of substitution rates and tMRCAs^1^.

**Subgenotype**	tMRCA*(years)*	MRCA date*(year)*	HPD95%*(years)*	Rate*(s/s/y)*	HPD95%*(s/s/y)*
**F1b**	47	1966	(16–158)	7.4 x 10^−5^	1.3 x 10^−5^–1.5 x 10^−4^
**F4**	13	2000	(12–15)	8.9x 10^−4^	4.1 x 10^−4^–1.7 x 10^−3^

^**1**^Median substitution rates and tMRCAs corresponding to the Bayesian coalescent analysis performed under the UCLN-BSP model by calibration with time-stamped sequences for the complete genome of HBV subgenotypes F1b and F4. Here s/s/y: substitution sites per year.

## Discussion

The study of the distribution of the HBV genotypes in the symptomatic acute course of infection provides information about their current regional circulation. In this study, we provide new epidemiological data on the HBV subgenotypes in acute symptomatic infections in Buenos Aires, Argentina. The 55 complete genome sequences included in this work represent about 25% of all complete sequences reported as HBV acute infections that are currently available on the GenBank. Particularly, they correspond to all available full length sequences of primary infections caused by subgenotypes F1b and F4.

The distribution of the described subgenotypes could be a reflection of the demographic history of the region, but it might also be consequence of the intrinsic biological features of the different strains. Moreover, the genotype distribution constitutes valuable information since different (sub)genotypes may present different clinical evolution.

In this work, the phylogenetic analysis grouped the full-length genome sequences as genotypes: F (70.9%), A (23.6%) and D (1.8%). The high prevalence of gF in acute infections from the metropolitan area of Buenos Aires and the differential distribution of genotypes according to the acute or chronic course of the infection were previously reported [15]. In that study, the genotype distribution for the acute infections was genotype F: 65.2%, genotype A: 30.4% and genotype D: 4.3%; but for the chronic infections, it resulted practically even (genotype F: 36.6%, genotype A: 26.8% and genotype D: 31.7%). Then, genotype F and genotype D appear as the most and the less prevalent in acute infections, respectively. However, subgenotype distribution was not analyzed.

At the subgenotype level, the analysis of complete genome sequences of our cohort grouped them as subgenotype F1b (52.7%), subgenotype A2 (21.8%), subgenotype F4 (18.2%) and a minority as subgenotype A1, B2, D3 and F2a (1.8% each).

In the previous study by González López Ledesma et al. [11], these samples have already been partially characterized, based on the analysis of S and BCP/preC regions, and similar results were found in acute infections. The same study has shown that the subgenotype distribution differs between acute and chronic infections, with the latter being distributed as follows: subgenotype F1b (25.6%), subgenotype A2 (17.8%), subgenotype F4 (20.2%) and genotype D (28.5%).

In summary, the results of the comparative analysis of acute versus chronic infections of these studies showed that subgenotypes A2 and F4 presented similar values in both groups, whereas subgenotype F1b and genotype D exhibit an opposite behavior (being subgenotype F1b more prevalent in acute than in chronic infections, while genotype D behaved otherwise).

This differential distribution could be explained at least by two non-mutually exclusive hypotheses.

At first, epidemiological changes in the population, such as population migrations, might represent inflexion points for viral genotype distribution, allowing the introduction of new viral strains into a given area. This was previously observed in Japan [20] and more recently in Italy, were the incidence of acute hepatitis B mostly sustained by genotype D had significantly decreased in the last decades, but the new HBV strains introduced through immigrant populations from countries with a higher endemicity constitute a new emergency [21]. Therefore, the recorded migrations from the Northern provinces of Argentina and from the neighboring countries to the metropolitan region since the 40s to the present might have impacted in the current genotype distribution leading to a primacy of genotype F, particularly subgenotype F1b [22].

On the other hand, the intrinsic biological features of the different viral genotypes might also be implicated in the biased distribution of (sub)genotypes between acute and chronic infections. Different studies have demonstrated the existence of dissimilar characteristics between the different viral types, particularly in relation to the HBeAg seroconversion-rate [9,13,23,24]. Recently, based on a differential distribution of subgenotypes in chronic HBeAg and antiHBe infections, it has been proposed that subgenotype F1b presents a lower seroconversion-rate when compared with subgenotype F4 and genotype D (14), although no longitudinal studies are available to confirm this proposal. The loss of HBeAg during chronic HBV infection is usually associated with a decrease in viral load, reducing the probability of transmission. Therefore, taking into account that most of the acute infections would arise from chronic HBeAg-positive cases [25], those genotypes or subgenotypes that show a later seroconversion of the HBeAg would be overrepresented in acute scenario, as is the case of subgenotype F1b.

The high prevalence of subgenotype F1b in the current infections might also have clinical implications for future chronic patients, given that it has been associated with a worse clinical outcome [13,15,26].

The differential behavior in the course of infection might impact the viral population dynamics [27,28]. The differences in the seroconvertion-rate between subgenotype F1b and subgenotype F4, and their implication in viral transmission, support a deeper analysis of the dynamics of those viral populations. The coalescence analysis showed that after a diversification process started in the 80s, the current sequences of subgenotype F1b were grouped in at least four highly supported lineages, whereas subgenotype F4 revealed a more limited diversification pattern with most lineages without offspring in the present. Even if these results might be influenced by the low number of subgenotype F4 isolates, they might also mirror the consequence of the above mentioned delayed HBeAg seroconversion event of subgenotype F1b, which would increase its probability of transmission and therefore, its higher incidence in the acute scenario.

In addition, almost no mutants involved in diagnostic fails, immune evasion or antiviral resistance were found, suggesting a low potential impact of these mutants in our epidemiologic situation.

In summary, the distribution of genotypes in the acute course of infections could be an expression of the genotypes being currently transmitted into new hosts. The observed predominance of subgenotype F1b in the acute infections might be related to recent internal migrations or to the intrinsic biological features of this subgenotype, which would increase its probability of transmission. More importantly, these results might be of clinical relevance, since the above mentioned subgenotype has been already associated with a more severe clinical course of infection.

## Supporting Information

S1 Table Primers employed to obtain the complete genome sequences. (DOCX)

S2 Table Data set of the acute isolates. (DOCX)

## Abbreviations

HBV: hepatitis B virus
HBeAg: hepatitis e antigen
BCP: basal core promotor region
preC: precore gene

## References

[1] Holmes EC. Evolutionary history and phylogeography of human viruses. Annu Rev Microbiol. 2008;62: 307–328. 10.1146/annurev.micro.62.081307.162912 18785840

[2] Kilpatrick a. M, Daszak P, Goodman SJ, Rogg H, Kramer LD, Cedeño V, et al Predicting Pathogen Introduction: West Nile Virus Spread to Galápagos. Conserv Biol. 2006;20: 1224–1231. 10.1111/j.1523-1739.2006.00423.x 16922238

[3] Mizokami M, Orito E, Ohba K, Ikeo K, Lau J, Gojobori T. Constrained evolution with respect to gene overlap of hepatitis B virus. J Mol Evol. 1997;44: s83–90. 9071016 10.1007/pl00000061

[4] Torres C, Blanco Fernández MD, Flichman DMM, Campos RH, Mbayed VA, Fernández MDB, et al Influence of overlapping genes on the evolution of human hepatitis B virus. Virology. 2013;441: 40–8. 10.1016/j.virol.2013.02.027 23541083

[5] Zhou Y, Holmes EC. Bayesian estimates of the evolutionary rate and age of hepatitis B virus. J Mol Evol. 2007;65: 197–205. 10.1007/s00239-007-0054-1 17684696

[6] Shi W, Zhang Z, Ling C, Zheng W, Zhu C, Carr MJ, et al Hepatitis B virus subgenotyping: History, effects of recombination, misclassifications, and corrections. Infect Genet Evol. Elsevier B.V.; 2013;16: 355–361. 10.1016/j.meegid.2013.03.021 23538336

[7] Tatematsu K, Tanaka Y, Kurbanov F, Sugauchi F, Mano S, Maeshiro T, et al A genetic variant of hepatitis B virus divergent from known human and ape genotypes isolated from a Japanese patient and provisionally assigned to new genotype J. J Virol. 2009;83: 10538–10547. 10.1128/JVI.00462-09 19640977 PMC2753143

[8] Kurbanov F, Tanaka Y, Mizokami M. Geographical and genetic diversity of the human hepatitis B virus. Hepatol Res. 2010;40: 14–30. 10.1111/j.1872-034X.2009.00601.x 20156297

[9] Lin C, Kao J-H. The clinical implications of hepatitis B virus genotype: Recent advances. J Gastroenterol Hepatol. 2011;26 Suppl 1: 123–30. 10.1111/j.1440-1746.2010.06541.x 21199523

[10] Rehermann B, Nascimbeni M. Immunology of hepatitis B virus and hepatitis C virus infection. Nat Rev Inmunol. 2005;5: 215–229. 10.1038/nri1573 15738952

[11] González López Ledesma MM, Mojsiejczuk LN, Rodrigo B, Sevic I, Mammana L, Galdame O, et al Hepatitis B Virus Genotype Distribution and Genotype-Specific BCP/preCore Substitutions in Acute and Chronic Infections in Argentina. PLoS One. 2015;10: e0121436 10.1371/journal.pone.0121436 25822666 PMC4378996

[12] Hayashi K, Katano Y, Takeda Y, Honda T, Ishigami M, Itoh A, et al Association of hepatitis B virus subgenotypes and basal core promoter/precore region variants with the clinical features of patients with acute hepatitis. J Gastroenterol. 2008;43: 558–564. 10.1007/s00535-008-2197-2 18648743

[13] Livingston SE, Simonetti JP, Bulkow LR, Homan CE, Snowball MM, Cagle HH, et al Clearance of Hepatitis B e Antigen in Patients With Chronic Hepatitis B and Genotypes A, B, C, D, and F. Gastroenterology. 2007;133: 1452–1457. 10.1053/j.gastro.2007.08.010 17920063

[14] Mayerat C, Mantegani A, Frei P-C. Does hepatitis B virus (HBV) genotype influence the clinical outcome of HBV infection? J Viral Hepat. 1999;6: 299–304. 10607244 10.1046/j.1365-2893.1999.00174.x

[15] Pezzano SC, Torres C, Fainboim H a, Bouzas MB, Schroder T, Giuliano SF, et al Hepatitis B virus in Buenos Aires, Argentina: genotypes, virological characteristics and clinical outcomes. Clin Microbiol Infect. 2011;17: 223–31. 10.1111/j.1469-0691.2010.03283.x 20545965

[16] Hall TA. BioEdit: a user-friendly biological sequence alignment editor and analysis program for Windows 95/98/NT. Nucleic Acids Symp Ser. 1999;41: 95–98.

[17] Guindon S, Gasguel O. A Simple, Fast, and Accurate Algorithm to Estimate Large Phylogenies by Maximum Likelihood. Syst Biol. 2003;52: 696–704. 10.1080/10635150390235520 14530136

[18] Darriba D, Taboada GL, Doallo R, Posada D. jModelTest 2: more models, new heuristics and parallel computing. Nat Methods. 2012;9: 772 10.1038/nmeth.2109 PMC4594756 22847109

[19] Drummond AJ, Rambaut A. BEAST: Bayesian evolutionary analysis by sampling trees. BMC Evol Biol. 2007;7: 214 10.1186/1471-2148-7-214 17996036 PMC2247476

[20] Kobayashi M, Ikeda K, Arase Y, Suzuki F, Akuta N, Hosaka T, et al Change of Hepatitis B Virus Genotypes in Acute and Chronic Infections in Japan. J Med Virol. 2008;80: 1880–1884. 10.1002/jmv.21309 18814241

[21] Sagnelli C, Ciccozzi M, Pisaturo M, Presti A Lo, Cella E, Coppola N, et al The impact of viral molecular diversity on the clinical presentation and outcome of acute hepatitis B in Italy. 2015;45: 137–147. 25915056

[22] Torres C, Leone FGP y, Pezzano SC, Mbayed VA, Campos RH. New perspectives on the evolutionary history of hepatitis B virus genotype F. Mol Phylogenet Evol. Elsevier Inc.; 2011;59: 114–122. 10.1016/j.ympev.2011.01.010 21296172

[23] Sheldon J, Rodès B, Zoulim F, Bartholomeusz a, Soriano V, Rode B. Mutations affecting the replication capacity of the hepatitis B virus. J Viral Hepat. 2006;13: 427–34. 10.1111/j.1365-2893.2005.00713.x 16792535

[24] Tong S, Kim K, Chante C, Wands J, Li J. Hepatitis B Virus e Antigen Variants. Int J Med Sci. 2005;2: 2–7. 15968333 10.7150/ijms.2.2 PMC1142218

[25] Milich D, Liang TJ. Exploring the biological basis of hepatitis B e antigen in hepatitis B virus infection. Hepatology. 2003;38: 1075–86. 10.1053/jhep.2003.50453 14578844

[26] Sánchez-Tapias JM, Costa J, Mas A, Bruguera M, Rodés J. Influence of hepatitis B virus genotype on the long-term outcome of chronic hepatitis B in western patients. Gastroenterology. 2002;123: 1848–56. 10.1053/gast.2002.37041 12454842

[27] Grenfell BT, Pybus OG, Gog JR, Wood JLN, Daly JM, Mumford J a, et al Unifying the epidemiological and evolutionary dynamics of pathogens. Science. 2004;303: 327–32. 10.1126/science.1090727 14726583

[28] Zehender G, Ebranati E, Gabanelli E, Shkjezi R, Lai A, Sorrentino C, et al Spatial and temporal dynamics of hepatitis B virus D genotype in Europe and the Mediterranean Basin. PLoS One. 2012;7: e37198 10.1371/journal.pone.0037198 22662136 PMC3360700

