# Supplementary material for: Analysis of the Molecular Evolution of Hepatitis B Virus Genotypes in Symptomatic Acute Infections in Argentina
Source: PLoS One. 2016 Jul 19;11(7):e0159509. doi: 10.1371/journal.pone.0159509 (PMC4951016; doi:10.1371/journal.pone.0159509)
Supplement: S1 Table — (DOCX) [file pone.0159509.s001.docx]

**Supplementary Table S1:** Primers employed to obtain the complete genome sequences.

| Fragment | PCR Round | Fragment length | Primer name | Sense | Sequence 5'-3' | Position (nt) |
| --- | --- | --- | --- | --- | --- | --- |
| 1 | 1st | 758 pb | HBV27 | F | CTG CTG GTG GCT CCA GTT C | 57-75 |
|  |  |  | HBV26 | R | AGA AAA TTG GTA ACA GMG GYA | 814-794 |
|  | 2nd | 585 pb | HBV29 | F | GCG GKG TKT TTC TTG TTG ACA A | 205-226 |
|  |  |  | HBV28 | R | GGG ACT CAA GAT GYT GYA CAG | 789-769 |
| 2 | 1st | 884 pb | HBV45 | F | CCG TTT CTC CTG GCT CAG TTT | 660-680 |
|  |  |  | HBV76 | R | CCG CGT AAA GAG AGG TGC GC | 1543-1524 |
|  | 2nd | 561 pb | HBV19 | F | ATT TGT TCA GTG GTG CGT AGG | 688-708 |
|  |  |  | HBV20 | R | AAA GGT TCC ACG CAT GCG CT | 1248-1229 |
| 3 | 1st | 632 pb | HBV21 | F | CTG TGC CAA GTG TTT GCT GA | 1171-1190 |
|  |  |  | HBV66 | R | CAG ACC AAT TAT GCC TAC A | 1802-1782 |
|  | 2nd | 506 pb | HBV71 | F | TGC CAA GTG TTT GCT GAC GC | 1175-1194 |
|  |  |  | HBV22 | R | TCC TGA AAG TCC AAG AGT CCT C | 1680-1659 |
| 4 | 1st | 875 pb | HBV73 | F | ATG GAG ACC ACC GTG AAC GC | 1608-1627 |
|  |  |  | HBV2 | R | CCC ACC TTA TGA GTC CAA GG | 2482-2463 |
|  | 2nd | 773 pb | HBV73 | F | ATG GAG ACC ACC GTG AAC GC | 1608-1627 |
|  |  |  | HBV54 | R | GAG TTC TTC TTC TAG GGG ACC TG | 2381-2359 |
| 5 | 1st | 830 pb | HBV63 | F | AGT GTG GAT TCG CAC TCC T | 2269-2287 |
|  |  |  | HBV84 | R | TGAGCCTGAGGGCTCCAYCC | 3098-3079 |
|  | 2nd | 683 pb | HBV63 | F | AGT GTG GAT TCG CAC TCC T | 2269-2287 |
|  |  |  | HBV86 | R | GTTGAAGTCCCARTCKGGA | 2951-2933 |
| 6 | 1st | 1162 pb | HBV17 | F | TGC ATA CTT TAT GGA AGG CGC | 2749-2769 |
|  |  |  | HBV31 | R | TGC ACT AGT AAA CTG AGC CA | 689-670 |
|  | 2nd | 863 pb | HBV17 | F | TGC ATA CTT TAT GGA AGG CGC | 2749-2769 |
|  |  |  | HBV60 | R | AAC GCC GCA GAC ACA TCC A | 391-373 |
