# Supplementary material for: Analysis of the Molecular Evolution of Hepatitis B Virus Genotypes in Symptomatic Acute Infections in Argentina
Source: PLoS One. 2016 Jul 19;11(7):e0159509. doi: 10.1371/journal.pone.0159509 (PMC4951016; doi:10.1371/journal.pone.0159509)
Supplement: S2 Table — (DOCX) [file pone.0159509.s002.docx]

**Supplementary Table S2:** Data set of the acute isolates

| **GenBank accesion number** | **Isolate** | **Gender** | **Age** | **Subgenotype** | **Country (City)** | **Isolation date** | **Origin of the sample** |
| --- | --- | --- | --- | --- | --- | --- | --- |
| KJ843183 | Ag22 | F | 38.2 | A1 | Argentina  (Buenos Aires) | 29-Jun-2011 | Acute infection |
| KJ843166 | Ag04 | M | 67.3 | A2 | Argentina  (Buenos Aires) | 15-Apr-2013 | Acute infection |
| KJ843172 | Ag10 | M | 69.0 | A2 | Argentina  (Buenos Aires) | 28-Apr-2010 | Acute infection |
| KJ843173 | Ag11 | F | 46.8 | A2 | Argentina  (Buenos Aires) | 08-Feb-2013 | Acute infection |
| KJ843182 | Ag21 | F | 45.1 | A2 | Argentina  (Buenos Aires) | 16-Jan-2012 | Acute infection |
| KJ843184 | Ag23 | M | 22.7 | A2 | Argentina  (Buenos Aires) | 17-Apr-2013 | Acute infection |
| KJ843186 | Ag25 | M | 32.4 | A2 | Argentina  (Buenos Aires) | 13-Feb-2013 | Acute infection |
| KJ843188 | Ag27 | M | 34.3 | A2 | Argentina  (Buenos Aires) | 31-Aug-2012 | Acute infection |
| KJ843192 | Ag31 | M | 41.3 | A2 | Argentina  (Buenos Aires) | 02-Feb-2010 | Acute infection |
| KJ843214 | Ag55 | M | 34.0 | A2 | Argentina  (Buenos Aires) | 19-Jan-2001 | Acute infection |
| KJ843215 | Ag56 | M | 41.0 | A2 | Argentina  (Buenos Aires) | 18-May-2004 | Acute infection |
| KJ843216 | Ag57 | F | 48.9 | A2 | Argentina  (Buenos Aires) | 09-May-2003 | Acute infection |
| KJ843217 | Ag62 | F | n/a | A2 | Argentina  (Buenos Aires) | 14-Dec-2013 | Acute infection |
| KJ843218 | Ag63 | M | n/a | A2 | Argentina  (Buenos Aires) | 20-Dec-2013 | Acute infection |
| KJ843162 | Ag72 | M | 41.0 | A2 | Argentina  (Buenos Aires) | n/a | Acute infection |
| KJ843165 | Ag03 | F | 22.7 | B2 | Argentina  (Buenos Aires) | 08-Mar-2012 | Acute infection |
| KJ843187 | Ag26 | F | 31.5 | D3 | Argentina  (Buenos Aires) | 09-Nov-2011 | Acute infection |
| FJ657521 | BA111 | M | 56.6 | F1b | Argentina  (Buenos Aires) | 05-Dec-2002 | Acute infection |
| FJ657523 | BA112 | M | 30.3 | F1b | Argentina  (Buenos Aires) | 24-May-2001 | Acute infection |
| FJ657524 | BA113 | M | 26.0 | F1b | Argentina  (Buenos Aires) | 15-Jan-2001 | Acute infection |
| KJ843163 | Ag01 | M | 47.5 | F1b | Argentina  (Buenos Aires) | 30-Mar-2011 | Acute infection |
| KJ843164 | Ag02 | M | 50.2 | F1b | Argentina  (Buenos Aires) | 04-Jan-2013 | Acute infection |
| KJ843167 | Ag05 | M | 49.1 | F1b | Argentina  (Buenos Aires) | 09-Aug-2011 | Acute infection |
| KJ843168 | Ag06 | M | 38.3 | F1b | Argentina  (Buenos Aires) | 13-Jan-2010 | Acute infection |
| KJ843169 | Ag07 | F | 69.2 | F1b | Argentina  (Buenos Aires) | 13-Mar-2012 | Acute infection |
| KJ843170 | Ag08 | M | 69.3 | F1b | Argentina  (Buenos Aires) | 02-Mar-2012 | Acute infection |
| KJ843171 | Ag09 | F | 48.6 | F1b | Argentina  (Buenos Aires) | 03-Apr-2013 | Acute infection |
| KJ843174 | Ag12 | M | 31.7 | F1b | Argentina  (Buenos Aires) | 13-Nov-2010 | Acute infection |
| KJ843176 | Ag14 | F | 69.5 | F1b | Argentina  (Buenos Aires) | 02-Sep-2012 | Acute infection |
| KJ843177 | Ag15 | F | 49.7 | F1b | Argentina  (Buenos Aires) | 23-Apr-2012 | Acute infection |
| KJ843178 | Ag16 | M | 27.6 | F1b | Argentina  (Buenos Aires) | 18-Mar-2013 | Acute infection |
| KJ843179 | Ag17 | M | 25.7 | F1b | Argentina  (Buenos Aires) | 27-Apr-2011 | Acute infection |
| KJ843180 | Ag18 | M | 46.0 | F1b | Argentina  (Buenos Aires) | 17-Aug-2012 | Acute infection |
| KJ843181 | Ag20 | M | 27.2 | F1b | Argentina  (Buenos Aires) | 02-Oct-2012 | Acute infection |
| KJ843190 | Ag29 | M | 36.5 | F1b | Argentina  (Buenos Aires) | 23-Oct-2012 | Acute infection |
| KJ843193 | Ag32 | M | 47.8 | F1b | Argentina  (Buenos Aires) | 07-Jan-2013 | Acute infection |
| KJ843194 | Ag33 | M | 66.3 | F1b | Argentina  (Buenos Aires) | 24-Apr-2013 | Acute infection |
| KJ843195 | Ag34 | M | 27.6 | F1b | Argentina  (Buenos Aires) | 30-Apr-2013 | Acute infection |
| KJ843196 | Ag35 | F | 43.3 | F1b | Argentina  (Buenos Aires) | 04-Sep-2003 | Acute infection |
| KJ843197 | Ag36 | M | 29.0 | F1b | Argentina  (Buenos Aires) | 09-Apr-2002 | Acute infection |
| KJ843198 | Ag37 | M | 22.6 | F1b | Argentina  (Buenos Aires) | 22-Dec-2003 | Acute infection |
| KJ843199 | Ag38 | M | 40.5 | F1b | Argentina  (Buenos Aires) | 25-Nov-2004 | Acute infection |
| KJ843200 | Ag39 | M | 28.6 | F1b | Argentina  (Buenos Aires) | 20-Nov-2003 | Acute infection |
| KJ843201 | Ag41 | M | 36.9 | F1b | Argentina  (Buenos Aires) | 09-Apr-2003 | Acute infection |
| KJ843202 | Ag42 | F | 25.7 | F1b | Argentina  (Buenos Aires) | 17-May-2002 | Acute infection |
| KJ843203 | Ag43 | M | 16.9 | F1b | Argentina  (Buenos Aires) | 02-May-2001 | Acute infection |
| KJ843204 | Ag44 | M | 34.2 | F1b | Argentina  (Buenos Aires) | 12-Aug-2003 | Acute infection |
| KJ843205 | Ag45 | M | 26.1 | F1b | Argentina  (Buenos Aires) | 09-Nov-2001 | Acute infection |
| KJ843206 | Ag46 | M | 42.4 | F1b | Argentina  (Buenos Aires) | 30-Sep-2003 | Acute infection |
| KJ843191 | Ag30 | M | 30.0 | F2a | Argentina  (Buenos Aires) | 02-May-2012 | Acute infection |
| FJ657522 | BA116 | M | 36.3 | F4 | Argentina  (Buenos Aires) | 07-Apr-2003 | Acute infection |
| KJ843175 | Ag13 | M | 17.0 | F4 | Argentina  (Buenos Aires) | 30-Sep-2012 | Acute infection |
| KJ843185 | Ag24 | M | 41.9 | F4 | Argentina  (Buenos Aires) | 18-Sep-2010 | Acute infection |
| KJ843189 | Ag28 | M | 39.8 | F4 | Argentina  (Buenos Aires) | 11-Jul-2012 | Acute infection |
| KJ843207 | Ag47 | M | 27.4 | F4 | Argentina  (Buenos Aires) | 31-Oct-2001 | Acute infection |
| KJ843208 | Ag48 | M | 41.9 | F4 | Argentina  (Buenos Aires) | 28-Feb-2001 | Acute infection |
| KJ843209 | Ag49 | M | 62.1 | F4 | Argentina  (Buenos Aires) | 27-Jul-2012 | Acute infection |
| KJ843210 | Ag50 | M | 39.0 | F4 | Argentina  (Buenos Aires) | 01-Nov-2000 | Acute infection |
| KJ843211 | Ag51 | M | 27.2 | F4 | Argentina  (Buenos Aires) | 15-Apr-2004 | Acute infection |
| KJ843212 | Ag52 | M | 48.7 | F4 | Argentina  (Buenos Aires) | 02-Feb-2004 | Acute infection |
| KJ843213 | Ag53 | M | 34.1 | F4 | Argentina  (Buenos Aires) | 25-Apr-2003 | Acute infection |
